# On Excessive Hemorrhage from the Extraction of Teeth, with Cases

**Published:** 1852-04

**Authors:** J. L. Levison

**Affiliations:** Brighton, England.


### ARTICLE III.

### *On Excessive Hemorrhage from the Extraction of Teeth, with Cases.* BY J. L. LEVISON, Brighton, England.

THOSE who are acquainted with the diseases connected with the sanguiferous system, are aware that extreme hemorrhages may result from many causes: from accidents; from debility in the coats of the arteries, as in purpura hemorrhagica; from a state of plethora; from surgical operations; from unequal circulation; as a symptom of scorbutus; and lastly, from the extraction of teeth. The latter, which will form the subject of this communication, is not exactly similar, either in the conditions of health under which it may occur, differing in different cases; nor can it be always distinguished from the preceding kinds; we therefore propose to detail some instances, with the remarks they suggest, and the treatment found to be the best to be depended upon; and afterwards add the substance of the information interspersed in medical works. For though the occurrence is rare of excessive bleeding after the extraction of teeth, still, when it does occur in the practice of the surgeon dentist, it is important that he should know what is best to be done under such exigencies. And it is just because there are but few of the emergent cases recorded, that those now to be submitted may be worthy of attention, and may induce others to communicate similar instances, so that ultimately some practical inferences may be given explanatory of the physiological and chemical condition of the blood in persons of this diathesis. That this notion is neither unreasonable nor uncalled for, will be admitted, when we state that nosologists have not given a name to this idiopathic form of disease, to distinguish it from other forms of excessive bleeding:* much less have they attempted to define the symptoms by which it is indicated.

From the consideration that every one practicing in surgery or medicine, is more or less indebted to preceding observers, it becomes, therefore, a principle which cannot be too often enforced, that all facts, whether connected with any special form of disease, or as evidence of distinct modifications, that it is a duty to bring them to the common stock, whether they may appear of sufficient interest or not, either in confirming previous conditions of disease, or in elucidating phenomena which had heretofore been obscure. And farther, in justification of the feeling which prompted this communication, the writer could not find in any of the medical dictionaries, (Hooper's, Parr's, &c.) any allusion to this special form of disease or particular idiosyncrasy, except a short notice in Cooper's Surgical Dictionary,† which is given under the generic phrases "Of BLEEDING DIATHESIS," or hemorrhagic tendency. Before then citing our own cases, it may be further premised that all that is known, on the conditions of health and disease, has been obtained by many observations on individuals, comparing their normal states of bodily and mental functions; and their disordered conditions or symptoms, and in this way *data* have been accumulated so as now to enable practitioners to distinguish in their diagnoses, any resemblances, or special differences. But this has not been done in the disease which is treated of in this paper, and which it is hoped will be of some interest to the readers of the Journal of Dental Science.

* For example, hemoptysis, epistaxis, hemeatemesis, hematura, &c.

† See Supplement, note *a*.

The dangerous bleeding which often takes place after the extraction of a tooth, is, *sui-generis*, so far as its present history is to be depended on. Even in the cases to be detailed, there did not seem any well marked analogy in the conditions of health of the different patients, or in their physiognomical expression—but which are so well indicated in other forms of excessive bleeding. One thing seems certain, as confirmed by experience—that it is not only idiopathic, *but* hereditary.

Case 1.—During my residence at Birmingham, Joseph Hodgson, Esq., an eminent consulting surgeon, recommended a lady to me, to remove some diseased molar teeth. She appeared ill from continued suffering, and the injurious effects of broken rest. There was great palor and feebleness in her features, she seemed of a cachectic habit, and that want of stamina, so well expressed in popular language, "as not looking in good health." She was of a nervo-lymphatic temperament. When the teeth were removed, she bled freely, yet not so much as to alarm either the patient or myself. A simple styptic sufficed to stop the hemorrhage, by means of a piece of cotton dipped into the oil of turpentine, and pressed on the bleeding vessels.

The operation took place at 10 o'clock, A. M., and at four in the afternoon, I had a message brought by her man servant, "that the lady from whom I had extracted the teeth, had been bleeding ever since, and that she was in a very weak and dangerous state !" The messenger himself looked as pale as if he had been deputed to announce to me the death of his mistress.

Prepared with every means that might be required, I went off to do my best, armed with many potent compounds, tinct. *ferri, muriat, argentum nitratum*, &c. On my arrival at the hotel, I saw consternation in the faces of all ! An attendant showed me to the patient's bed room, in a hurried and nervous manner. She was lying on her bed in a prostrated condition—her face blanched and terror-stricken, but restless and irritable; with a pulse very low and feeble. I stopped the hemorrhage by forcing a stick of caustic (the argentum nitratum) to the bottom of each septum of the alveolii, and by crushing some of it and dissolving it in a little water, so as to be able to saturate some pieces of cotton wool, and so pressed it down, that each cavity was firmly plugged. Soon the bleeding ceased, and the patient fell into a sound sleep, and at the end of two hours awoke much refreshed, the bleeding having entirely ceased. From her, however, I learnt that on a previous occasion when she was obliged to lose a portion of a tooth, she had bled without any intermission for 35 hours, until she fainted, and the hemorrhage stopped of itself. She also told me that many of her family had the same peculiarity, particularly on her mother's side.

After these details, we may venture to affirm that no one would class the case of this lady as one of purpura hemorrhagica, as she was free from all extravasation of blood under the cuticle. There were neither spots nor patches, nor any hemorrhagic discharge from any other organs usually affected in this form of disease. Further—the bleeding of this patient cannot be considered as a symptom of scorbutus. For in this latter, depressing disorder, the gums often form a diagnostic sign of its presence, in which case they scent the air with a nauseating fetor. The case itself made me a little fidgetty, as there had been a fatal one just before, in the modern Athens, and which, with all the skill of the Edinburgh men to save the poor fellow, proved useless. (Read Lancet for the year 1841 or '2, in which the details are recorded.) My nervousness was in consequence of a paragraph that went the round of the papers, entitled "DEATH FROM THE EXTRACTION OF A TOOTH," and induced extreme dread in those who required the operation. Whilst the recollection of these things was still vivid in my memory, a surgeon, the late Alfred Jakes, Esq., of Birmingham, sent a young girl about eight years old, to have the first permanent molar tooth extracted. She was very pale and thin, and of a strumous habit—at the same time suffering from whooping cough. Mentally she was deficient, having small anterior lobes. Whilst in the few brief minutes I saw her, she displayed strong opposition, the symptoms of a more than ordinary combativeness and destructiveness, which produced a fit of coughing of such violence that she split the skin of the lower lip, when, literally a stream of thin limpid blood poured forth, and therefore, as I had not any ambition to kill the little termagant, I persuaded the mother to use a wash for the mouth, giving her a prescription, being very glad to get rid of such a patient.

In this instance, there were many symptoms which resembled scurvy—the fetor of the breath—the tumid state of the gums—the expression of the face—the general emaciation and debility, and hence the bleeding in this case from the weak state of the arteries, might be referred either to the scorbutus or to purpura hemorrhagica, as it might be regarded as spontaneous.

We shall find that the quantity and duration of the bleeding from the extraction of a tooth, are not to be indicated by the apparent health of the patient, as the following will testify.

CASE 2.—Mrs. L—— made a voyage from Jamaica to England, under an impression that the journey and the treatment by eminent medical men in her own native country, would restore her to health. Finding herself not so much improved, and requiring a tonic atmosphere, she was recommended to this "Queen of Watering Places," where I had been located since 1844. Some time in 1846 she consulted me for her little daughter, from whose mouth I extracted some few temporary teeth. The child bled profusely for a short time, but it soon ceased. One Saturday Mrs. L—— came to have one of her own teeth extracted, the second molar of the upper jaw. The tooth was small and the fangs short and thick, and she suffered very little from its removal. There was some bleeding, but not to any great excess. Mrs. L's person harmonized with her teeth, she was short and thin, somewhat pale, but with a good head, and an active nervous system. When she left, she said smilingly, "I am better off this time, than I anticipated." To me it was either a marvellous expression, or else a mode of showing her gratitude for the relief she had experienced. About eleven that same night, the mystery was solved. I received a note, written in pencil, telling me that Mrs. L—— had been bleeding all day, and was still doing so in great quantities, and requesting I would send something to stop it. Before an answer could be returned in the usual method of conveying it, I was at the residence, and in the bed room of the lady. She looked as she laid on the bed, like a piece of exquisitely white sculptured marble. Her hands and feet had already the coldness of death, and her voice was too feeble to hear! Though somewhat agitated at their neglecting to send for me before, I have reason to be grateful for my presence of mind. Putting my hand on her wrist, I could not perceive the slightest movement of her pulse. I called for wine, some sherry was brought to me. With my hand still on her wrist, I ordered the servant and her relations to raise her up a little, and to continue to give her wine until I bade them stop. She had had the sixth glass, when to my satisfaction, the pulse became manifest, and then, and then only, I turned to stop the bleeding, which the ordinary styptics failed to do. So I dipped a piece of cotton in the bi-chloride of zinc, undiluted, and pressed it on the bleeding vessels. The pain was great, as the tendency of this preparation is to destroy the bleeding vessels. She recovered and improved in health, and was often afterwards with me, as a patient, until her departure.

I have been rather particular in the details, in order to point out a fact, that if on my arrival, I had attempted to stop the bleeding, when the heart scarcely acted, in all probability a collapse would have taken place, and the patient would have died under my hands.

It may be worthy to note in this place, what has been hinted at before, that in all cases of this kind, which are recorded, incontestibly prove that it is hereditary. Such was the fact in cases Nos. 1 and 2, and also in the two now to be narrated.*

CASE 3.—One day during the summer of 1848, a lady, Miss W——, applied to me to extract for her a molar tooth on the lower jaw, and as she seemed very nervous on the subject, I endeavored to impress her with the fact that it would be comparatively a trifling operation, and that of but short duration. She replied, "I am not alarmed at the amount of pain, but for

* See also a case at the concluding portion of this paper.

the after consequences, as I am one of a family of *bleeders*. For whenever I have been forced to undergo this, or any even trifling operation, the surgeon has hitherto had a great amount of difficulty in stopping the frightful hemorrhage." After thanking her for the information, and assuring her that I would take means to prevent the consequences she anticipated, I extracted the tooth. The flow of blood was terrific, and although her features became pallid, and a clammy perspiration came over her, which is often a premonition of a state of *syncope*, yet the bleeding continued with great impetuosity. I therefore dipped small pieces of cotton wool into the bi-chloride of zinc, and forced them down the alveolar processes to the bottom of each cavity, and although a painful operation, by its immediately corrugating the lacerated arteries, the hemorrhage was arrested. But the plugs were permitted to remain with the cotton pressed down tight on them. The lady left me in good spirits, and continued, at my request, to retain the plugging for three days. At the end of that period, I removed them, and no ill consequences ensued.

In this instance, there was still less than in the preceding ones, to indicate such a peculiarity. The lady seemed in health, probably somewhat dyspeptic, but with that enduring temperament—the nervo-bilious.

About two months afterwards I was sent for to attend a brother of the above patient, the Rev. —— W——, and when I arrived at his lodgings, I found him in a state of great agitation as he had the family tendency, and said "that he dreaded the operation in consequence of the sufferings he endured on the last occasion, when he had had only a part of a tooth extracted. That he had been obliged to be three weeks under Sir Benjamin Brodie, and that at last that celebrated surgeon had to tie the carotid artery." It was, therefore, with no very pleasant presentiments, that I undertook the task—the detail being as follows.

CASE 4.—The Rev. —— W——, about twenty-four years of age, a nervo-lymphatic temperament, with some slight mixture of the sanguineous. He was curate of a very large parish, and had, during his clerical labors, caught some epidemic disease, which had reduced him very much. Besides which he had not long been married. So that he had suffered from mental excitement of various kinds, and had visited Brighton to recover his health. I extracted the tooth, a molar, in the upper jaw. It was about four o'clock on a Saturday afternoon. When it was out, the blood gushed forth in jets, so that in the first instance, as he was in such an irritable state of mind, I tried a strong styptic with compression, without having recourse to the more severe remedy, and the bleeding seemed stopped.

About 12 o'clock, that same night, I was aroused by violent knocking, and ringing, at my street door, and a messenger bellowed forth, as if he himself had been doomed to immolation, "the Rev. —— W—— is bleeding most profusely, and if you dont go immediately he'll die." When I entered the chamber of my patient, he was in bed, with a dressing gown on, and his head over a hand-basin, literally pouring out blood, which seemed very thin, but of a dark color. His face was flushed, his head hot, and his eyes bright and with a wild expression. Having given him an assurance that I would stop the bleeding, but that in doing so he would suffer great pain. He replied in a hurried, and rather weak manner, "*Pray then do it.*"

I then adopted the plan, dipping the cotton in the bi-chloride of zinc, and pressed it down on the bleeding vessels. This occasioned him great agony. He jumped out of bed, railing like a maniac, but I forced him back again to complete the plugging. The bleeding ceased. And after remaining all night with him, he was quiet, and had had no return of the hemorrhage. Dr. Peckford, a gentleman of high professional qualifications, attended Mr. W—— at the same time, and he suggested that he should have ice to his face, and only take cold slops, so as not to disturb the mouth by the motion of chewing food. The next and following days he continued without any return of the bleeding, for the application had caused a large clot to fill up the space of the tooth formerly and the one recently removed; but on Monday night I was again called up, "as he had recommenced bleeding," so said the messenger. On my arrival, I found him in the old attitude in bed, holding his head over a basin, in which some water was tinged with a small quantity of blood. I told him that this was indeed, "a false alarm," and on looking into the mouth, there was the clot inside, but he had rubbed the cuticle of the gum on the one side of it, and abraided the capillaries, but I painted the surface with a diluted solution of the bi-chloride of zinc, and immediately stopped the hemorrhage.

Assured by his medical attendant and myself that there was not any probability of a recurrence of the dangerous symptoms, he remained in bed quiet, taking the fluid diet, and he rapidly improved afterwards, the mouth healed, and under a tonic course, he recovered his health.

It may not be altogether gratuitous to inquire if we derived any *data* from the above cases, as to the chemical condition of the blood, to explain the phenomena, even if we cannot speculate on the causes of this peculiar affection. We may remark, incidentally, that in all the cases we have seen, there seems to be an abnormality of the blood, which appears to have an insufficiency of fibrine, and thus, for this reason, it does not form a *coagulum:* and this incapacity to form a *clot*, (which is nature's plug for lacerated and bleeding vessels,) may, in some measure, account for the excessive hemorrhage. And from the color of the blood, (though we had not the opportunity of proving this by any microscopical observations,) this would appear to confirm the latter speculation, the color being similar to the blood of persons to whom ether has been administered, for the purpose of producing anæsthesia, in which case the blood corpuscles are said to be ruptured. But whether, if such is the case in the excessive hemorrhage after the extraction of a tooth, it may still be either an idiopathic condition or the result of the nervous terror of the patient. But which, with our present experience, cannot be decided.

In the article "Blood," in Dr. Parr's London Medical Dictionary, (1809,) he says, "We find in scorbutic cases, and in some species of typhus, as Dr. Lind and MM. Parmentier and Deyaux have shown, that tenuity of blood is not occasioned by agitation,* but in the first, by a probable increase in the proportion of neutral salts with a deficiency of oxygen, and in the latter by a diminution of the proportion of fibrine or albumen; *a change which seems to be the constant consequence of debility.*"

That the important remark of Dr. Parr, which I have marked in *italics*, is worthy of being attended to, is manifest by the case of the little creature with the whooping cough. The stream of blood from whose lip being thin and of a rather darker color than arterial blood, showed a deficiency of oxygen; and in the instance of the Rev. T—— W——, it will be remembered, that after the vessels of the alveolus had been destroyed, and he injudiciously rubbed the gum near it, that even the small capillaries continued to bleed to a degree sufficient to excite the terror of the patient. So that there was a deficiency of fibrine, &c. inducing a fluid of the state of the blood. *(a)* It seems that the bleeding tendency in some of these cases, may arise in part from the extreme thinness of the arteries.

We will now cite a few incidental opinions on this subject, in order to show how indefinite the statements are, and how comparatively useless in treating similar cases.

* Dr. Parr is refuting the notion that fluidity in the arteries is induced by the motion within them.

*(a)* Under the head of "Hemorrhagic Tendency," the late excellent surgeon, Samuel Cooper, in his Surgical Dictionary says, "there appears to be an extraordinary tendency to profuse hemorrhage from very slight injuries." An instance of this kind has been recorded by Mr. Blagden, when a fatal hemorrhage arose from the extraction of a tooth. The patient was twenty-seven years of age, had had a tooth extracted when a boy, in consequence of which operation the bleeding continued twenty-one days from the socket, before it ceased. A very slight cut in the hand was also followed by an alarming bleeding, which could not be stopped by pressure, styptics or ligature, so that it became necessary to apply the *kali purum,* which succeeded. On having another carious tooth taken out, a profuse bleeding followed, which resisted the effects of styptics, caustic, and every means adopted to stop up the socket. The actual cautery was tried in vain. The dangerous condition of the patient seemed to leave no other

After the various means had been found futile, it appears to me, that though highly painful as the operation of destroying the lacerated arteries by the bi-chloride of zinc may be, yet when the excessive bleeding occurs after the tooth is extracted, that it is worthy of trial, and in my own cases never failed.

Mr. Cooper adds, "Several instances are recorded in which the males of a particular family had this hemorrhagic tendency, so that whether the bleeding arose spontaneously, or from a wound, it could not be suppressed, and their lives were lost." The author then gives references to the following works: Dict. des Science Med., Art, Vas, Rares; London Medical Repository, vol. iii, page 60; Krümer's Versüchen Einer Physiologie des Blutz, page 115, Lepzig, 1823; Journal des Progres, pour 1828, &c.

There was also a case reported in vol. 5 of the Medical Gazette for 1830, under the title "Hemorrhage from the extraction of a tooth, by John Kendrick, Esq., of London, in which he details the history of the case, which this summary embodies. "He was requested to see Mr. P——, a remarkably fine young man, of twenty-three years of age, who the day before had had a left molar of the upper jaw extracted by a dentist, a portion of the alveolar process having been removed with the tooth, and left a rather frightful cavity. On his arrival the patient was bleeding copiously, and he had done so for five hours previously to his seeing him. The cavity was filled with *coagulum, on the removal of which, the blood flowed with greater rapidity*—per saltem, which clearly indicated it proceeded from an artery."

This, I should have called bad practice, as we have seen that the *clot*, or coagulum, forms the natural plug to the bleeding vessels. Mr. Kendrick, however, informs us that he stopped the hemorrhage with the *tinctura ferri muriatis*, but that resource but that of tying the carotid artery, which was done by Sir Benjamin Brodie. But even this proceeding failed to arrest the hemorrhage, which proved fatal." [From the Med. Chir. Trans., vol. viii, page 224, London, 1817.] Vide Cooper's Surgical Dictionary, page 697, seventh edition.

it afterwards recommenced with redoubled violence, and the patient appeared in a perilous condition, but after trying other means, he finally succeeded in stopping the bleeding with lint dipped in strong alcohol."

The paper, which is very interesting as the record of a case, concludes thus, "An extraordinary feature is, that the patient was one of a numerous family in Norfolk, who in almost every instance, when a tooth had been extracted, they had been more or less subject to violent hemorrhage."

We cannot help remarking, that the particular conditions of health, seem not to offer any solution to the enigma of those special bleeding cases. Nor are we aided by the temperaments, as the *nervous*—the *nervo-lymphatic*—the *sanguineous*—and the *lymphatic*, are all liable to this peculiar diathesis. It must, therefore, be a matter of great importance, when it is discovered that not a styptic in general use can be depended on, that we may arrest the dangerous hemorrhagic flow with certainty by destroying the vessels with the bi-chloride of zinc.

In the lectures of the late excellent Joseph Fox, (who was the first English dentist whose practice was based on anatomical, physiological and pathological *data*,) merely incidentally alludes to the subject of this Essay. He says, "sometimes, after the extraction of a tooth, a considerable hemorrhage continues, the artery belonging to the tooth does not contract in form, being of a large size, a *coagulum* sufficiently to restrain the flow of blood—hence great quantities of blood may be lost. The best plan or mode of stopping the hemorrhage is by the application of pressure: a very fine piece of lint or cotton, dipped in turpentine or alcohol, should be pressed into the socket, over which a large compress of lint should be laid, which may be pressed firmly by the finger, and in closing the mouth by the teeth of the upper jaw. In this manner a hemorrhage may be soon restrained. Little or no benefit is derived from washing the gums with styptic remedies, as they cannot act on the mouth of the bleeding vessels, and therefore are ineffectual."

Bell, in his work "On the Anatomy and Physiology of the Teeth," makes some fine general remarks on this subject:

and though he has, in his treatise, frequently deprecated some of Mr. Fox's views, yet it will be obvious, that although he offers some recommendation in the treatment of this form of hemorrhage as original, it must be confessed that *his* plan differs little from that incidentally given by his predecessor. I shall quote a few passages.

"A very severe and dangerous hemorrhage occasionally follows the extraction of a tooth. That this is, in some cases, the result of a hemorrhagic tendency in the constitution, or in other words, of a want of contractile power in the coats of the arteries, seems clear, from the circumstances that some persons never have a tooth extracted without a considerable hemorrhage continuing for a long time afterwards." After speaking of many fatal cases being recorded, he adds—"When it is recollected that the bleeding vessel is situated at the very bottom of the alveolar cavity, it is astonishing that the following simple method of applying pressure in immediate contact with it, which I am about to recommend, should long ago have been universally adopted. I have seen very many severe cases, and some of which the patients had nearly sunk from loss of blood, but I have never seen one in which I have failed to stop the hemorrhage in a very few minutes, by the following process."

I must, however, tell Mr. Bell, that in the cases I have narrated, and in others I could have given, the flow of blood had been so excessive as to defeat all such deliberate modes of proceeding. His plan shall now be given. "*The bleeding alveolus is first of all ascertained, the coagulum carefully removed from it, and the interior cleansed with a bit of lint, and by rinsing the mouth with warm water.* A strip of lint of sufficient length being torn off, one extremity is then introduced by means of a curved instrument, and gradually pressed down from time to time, until it reaches above the cavity, then a compress of lint is laid upon so thick as to be pressed upon by the antagonist teeth. The mouth is to be carefully closed, so as to retain the plug in its place, and kept constantly and firmly pressed against the bottom of the alveolar cavity."

We have said before, what we now repeat, that the coagulum is nature's plug, and hence in even such an extreme case as that of the Rev. T—— W——, I did not attempt to clean out the socket, or to wipe it out clean with lint, because the flow of blood was like a rapid running tap of water.

There seems to me little novelty in the plan recommended by Bell, because no one, except a dolt head, would attempt to press on a large and broad piece of lint into a narrow cavity: he would be guided, even the first time, by the process used in stopping a tooth with gold. Simple pressure may answer in some cases, as I recollect that a student in one of the London hospitals adopted a very novel, yet efficient plan for this purpose. A man had had a tooth extracted. The bleeding was excessive. Every kind of styptic then in general use was tried, and also the actual cautery, but the hemorrhage could not be stopped. The patient was sinking when a student of the hospital, with great presence of mind asked for the tooth. There was some trouble and delay in finding it, and when it was obtained, he replaced it into the alveolar processes of the patient, urged him to forcibly close the jaws together, and the bleeding vessel was thus effectually closed.

It appears that much of the obscurity in the peculiar affection we have commented on, arises from the phraseology, in which it is described, being so indefinite. It being confounded with other diseases; for example, in vol. 1 of the Lancet, for 1844, page 423, there is a case under the title of Hemorrhagic Diathesis, which proved to be "spontaneous bleeding from the scalp," and vol. 2, page 70, for 1845, there is a case of true purpura hemorrhagica, under a similar announcement.

In conclusion, if our essay, imperfect as it is, will tend to stimulate every dental practitioner to note all particulars of such cases; the state of health—modes of living—occupation—temperament—locality—and the chemical condition of the blood, and whether the affection was idiopathic, and whether it was hereditary, we might ultimately obtain so many valuable facts as to enable some future writer to give the pathology of the disease.

